# Electrical pulse stimulation parameters modulate N2a neuronal differentiation

**DOI:** 10.1038/s41420-024-01820-y

**Published:** 2024-01-25

**Authors:** Daniel Martín, Diego Ruano, Alberto Yúfera, Paula Daza

**Affiliations:** 1 Departamento de Biología Celular, Facultad de Biología, Universidad de Sevilla, Sevilla, Spain; 2 Instituto de Microelectrónica de Sevilla (IMSE), Consejo Superior de Investigaciones Científicas/Universidad de Sevilla, Sevilla, Spain; 3 Departamento de Bioquímica y Biología Molecular, Facultad de Farmacia, Universidad de Sevilla, Sevilla, Spain; 4 Instituto de Biomedicina de Sevilla (IBiS), Hospital Universitario Virgen del Rocío/Consejo Superior de Investigaciones Científicas/Universidad de Sevilla, Sevilla, Spain; 5 Departamento de Tecnología Electrónica, ETSII, Universidad de Sevilla, Sevilla, Spain

**Keywords:** Stem-cell differentiation, Cancer stem cells

## Abstract

Electrical pulse stimulation has been used to enhance the differentiation or proliferation of neuronal progenitor cells in tissue engineering and cancer treatment. Therefore, a comprehensive investigation of the effects caused by its parameters is crucial for improvements in those fields. We propose a study of pulse parameters, to allow the control of N2a cell line fate and behavior. We have focused on designing an experimental setup that allows for the knowledge and control over the environment and the stimulation signals applied. To map the effects of the stimulation on N2a cells, their morphology and the cellular and molecular reactions induced by the pulse stimulation have been analyzed. Immunofluorescence, rt-PCR and western blot analysis have been carried out for this purpose, as well as cell counting. Our results show that low-amplitude electrical pulse stimulation promotes proliferation of N2a cells, whilst amplitudes in the range 250 mV/mm–500 mV/mm induce differentiation. Amplitudes higher than 750 mV/mm produce cell damage at low frequencies. For high frequencies, large amplitudes are needed to cause cell death. An inverse relation has been found between cell density and pulse-induced neuronal differentiation. The best condition for neuronal differentiation was found to be 500 mV/mm at 100 Hz. These findings have been confirmed by up-regulation of the Neurod1 gene. Our preliminary study of the molecular effects of electrical pulse stimulation on N2a offers premonitory clues of the PI3K/Akt/GSK-3β pathway implications on the neuronal differentiation process through ES. In general, we have successfully mapped the sensitivity of N2a cells to electrical pulse stimulation parameters.

## Introduction

Electrical stimulation (ES) is a novel tool for controlling cell culture behavior and fate, where an electrical field (EF) is applied to a cell culture through electrodes. ES has been extensively investigated for its capacity to enhance or inhibit cell proliferation and differentiation. These capabilities make ES a powerful tool for tissue engineering and regenerative medicine, for example, in nerve regeneration [1, 2] or to improve implant osseointegration [3–5]. Although these applications are among the most common for ES, its potential extends beyond them; ES is being studied as a tool for intestinal disorder treatments [6, 7], muscular and cardiac dysfunctions [8, 9], and cancer [10]. The effects of ES on the culture or subject are regulated by the ES parameters and electrode setups. The three most important parameters of an electrical stimulation signal are waveform, amplitude and frequency, being another important factor, the stimulation time.

N2a is a mouse neuroblastoma cell line, commonly used to study neuronal differentiation [11–13], neuronal signaling pathways [14], neurite growth [15, 16] and cancer [17], among others. Neuroblastoma is a type of cancer of the nervous system that develops in cells that remain from the embryonic phase; it generally affects children. N2a cells are neural precursors and have the ability to differentiate, changing their morphology under the right conditions; when this happens, cells extend their cytoplasm forming neurites. Non-differentiated N2a cells have high proliferation, on the other hand, differentiated cells do not divide anymore. The ability to induce N2a cell differentiation and halt their proliferation holds promise for the treatment of neuroblastoma.

Direct current (DC) ES has previously been applied to N2a cells [18]; cells reacted by overgrowing their neurites with amplitudes in the range of 125–500 mV/mm. EF was applied vertically to the cell culture plate, carbon electrodes were placed one underneath and one on top, in the air. Induced differentiation has been found in neuronal precursor cells after applying a DC EF of 115 mV/mm [19]. Instead of a vertical EF, a horizontal one was applied; two opposing Ag/AgCl electrodes were introduced in the culture; this configuration is referred to as direct coupling. The same electrode configuration was used in [20], where a 30 mV/mm amplitude DC field, through nanotube-hydrogel composites, carried out a similar effect on PC12 cells. This cell line has also been found to differentiate under low-amplitude DC fields [21] using direct coupling with platinum wire electrodes. In [22] a pulsed field of 300 mV/mm–100 Hz induced neuronal differentiation of mouse neural stem cells. The authors of this work used an agar bridge electrode configuration. A similar setup was used in [23], were a DC field of 25–100 mV/mm induced morphological changes of N2a and BV2 cells.

While a wide variety of electrode configurations and stimulation protocols have been used to induce neuronal differentiation of precursor cells, most studies have not thoroughly explored the full spectrum of possibilities. In our investigation, we conducted a comprehensive exploration of two crucial ES parameters: amplitude and frequency. Additionally, we developed an experimental setup that enables precise control and comprehensive understanding of the stimulation applied to the culture. To examine the effects on N2a neuronal differentiation and proliferation, we studied a range of amplitudes and frequencies with a fixed waveform and stimulation time. Subsequently, a more in-depth analysis was performed under the best conditions. By employing this approach, we were able to generate a comprehensive map depicting the sensitivity of N2a neuronal differentiation to ES signal parameters, facilitating control over the fate and behavior of N2a cells. Moreover, we demonstrated that our ES protocol is sensed at the molecular level by inducing transcriptional expression and Akt-GSK-3β signaling pathway activation.

## Results

### ES amplitude and frequency effect on N2a fate

**Fig. 1:**
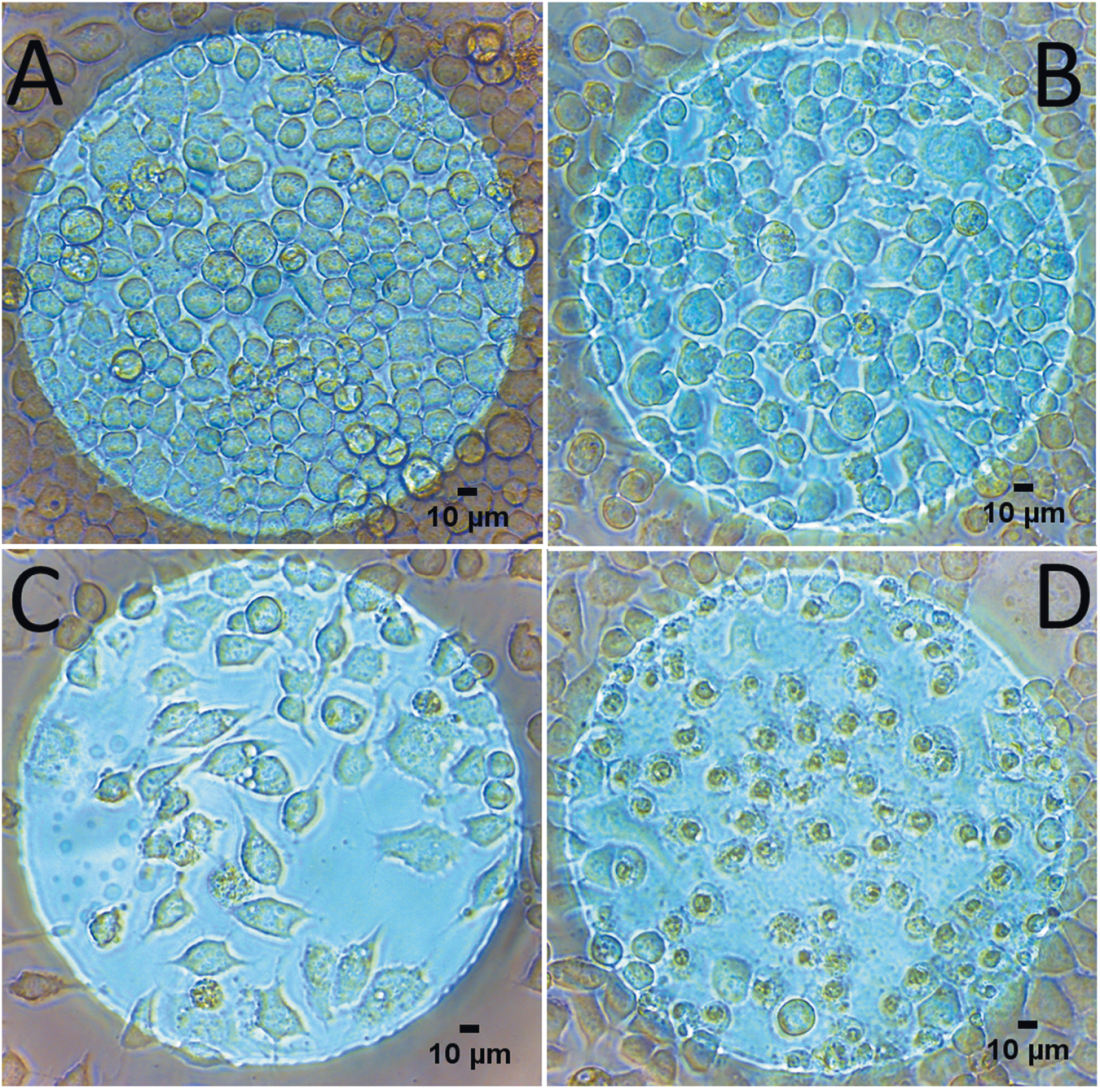
Examples of images used for counting. Images were taken on an inverted microscope directly on the 8W10E+ electrode plate with a 20× objective. **A** Control, **B** 125 mV–1 kHz. **C** 500 mV–100 Hz. **D** 1V-DC.

The main method to determine N2a differentiation was to analyze their morphology. Differentiated cells are more elongated and have extensions in the form of neurites; meanwhile, non-differentiated cells remained rounded. Figure 1 shows three examples of cells stimulated with different ES conditions and a control. Non-stimulated cells remained rounded and at high density (Fig. 1A), and as the amplitude of EF increased, cells started to differentiate and the density decreased (Fig. 1B, C). When the amplitude was too high, cells had their nucleus shrank and their membrane disrupted (Fig. 1D).

To study the effect of ES on N2a fate, we tested 42 different conditions, varying amplitude and frequency while maintaining ES time and waveform. The amplitude ranged between 125 mV/mm and 1.5 V/mm and the frequency between DC (0 Hz) and 100 kHz. Conditions were duplicated in two wells of the plate; one condition was always the control. Images of each electrode were taken and the cells on top of them were counted. Due to the quantity of data, results have been summarized on heat maps that represent the mean of the given result for every condition normalized to its control. The most interesting conditions will be further analyzed. Two maps were made: cell density and cell differentiation.

**Fig. 2:**
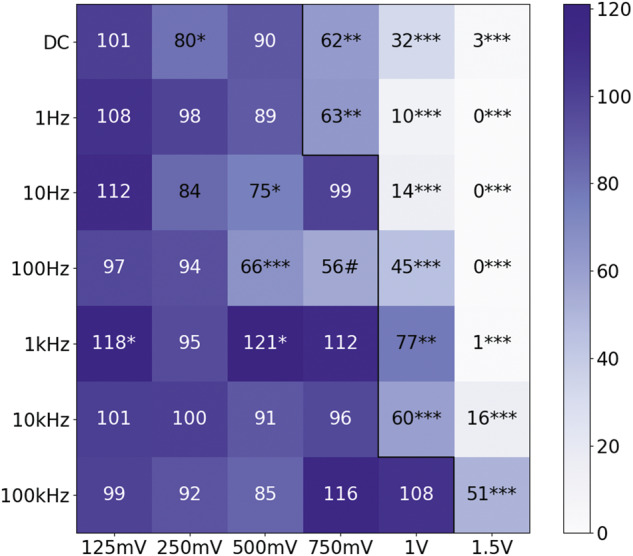
Cell density map. Each square on the map represents the mean number of cells per square millimeter normalized to the control condition of that experiment and expressed as a percentage. The black line separates conditions that caused apparent damage (right) from those that did not (left). Values over 85 are displayed on white to improve contrast. #: This point was interpolated as the mean of the closest amplitude values for the same frequency (500 mV and 1 V, 100 Hz). Voltages are equivalent to voltage/mm due to the distance between electrodes being 1 mm. (*p < 0.05; **p < 0.01; ***p < 0.001).

The first map (Fig. 2) corresponds to the cell density of N2a cells stimulated for 6 h under different conditions. The black line separates the conditions that caused apparent damage (right) from those that did not (left); this type of damage can be seen in Fig. 1D. Conditions with lower frequency seem to have a lower threshold for damage, this means that damage can be caused with lower amplitudes. 125 mV is the amplitude that promoted proliferation the most overall and 1 kHz the frequency that had the most effect on proliferation. Not taking 750 mV–100 Hz into account (interpolated point), 500 mV–100 Hz is the condition with the minimum cell density that did not cause apparent damage.

**Fig. 3:**
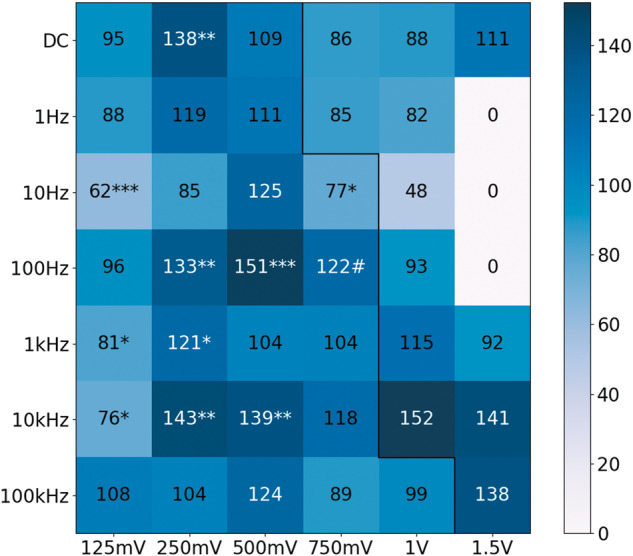
Differentiation percentage map. Each square on the map represents the mean percentage of differentiated cells normalized to the control condition of that experiment and expressed as a percentage. The black line separates conditions that caused apparent damage (right) from those that did not (left). Conditions that caused apparent damage have not been considered for statistical analysis. Values over 120 are displayed in white to improve contrast. #: This point was interpolated as the mean of the closest amplitude values for the same frequency (500 mV and 1 V, 100 Hz). Voltages are equivalent to voltage/mm due to the distance between electrodes being 1 mm. (*p < 0.05; **p < 0.01; ***p < 0.001).

The second result to discuss, was the cell differentiation percentage, represented in Fig. 3. As can be observed, 125 mV was the amplitude that promoted the least differentiation with values below those of the control. In general, it can be affirmed that 250 mV and 500 mV are the amplitudes that promoted differentiation the most, highlighting 250 mV-DC, 250 mV and 500 mV at 10 kHz and 250 mV and 500 mV at 100 Hz. The three frequencies previously mentioned seem to be the ones with better results.

**Fig. 4:**
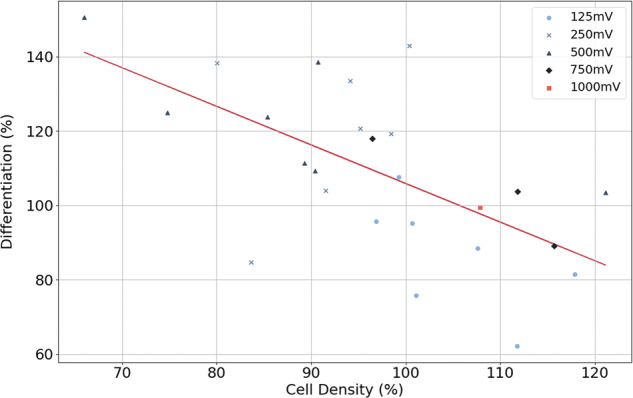
Relation between differentiation percentage and cell density. Points correspond to ES conditions as shown in Figs. 2 and 3. There is a linear regression line colored red that fits the blue points (*R*^2^ = 0.3879).

It is to be noted that the points in the second map (Fig. 3) that have a high value tend to have a lower value in the first map (Fig. 2) and that there is a trend where conditions that promoted differentiation reduced proliferation and vice versa. This seems to be correct, since differentiated cells do not divide anymore. Figure 4 shows a representation of the points of the two maps with respect to each other. The values, as shown on the maps, have been represented in a scatter plot with a red regression line. For example, the 500 mV–100 Hz condition can be seen in the upper left corner, with a cell density of 66 and a differentiation percentage of 151. The trend explained previously is clearly visible: with higher density comes less differentiation and vice versa.

**Fig. 5:**
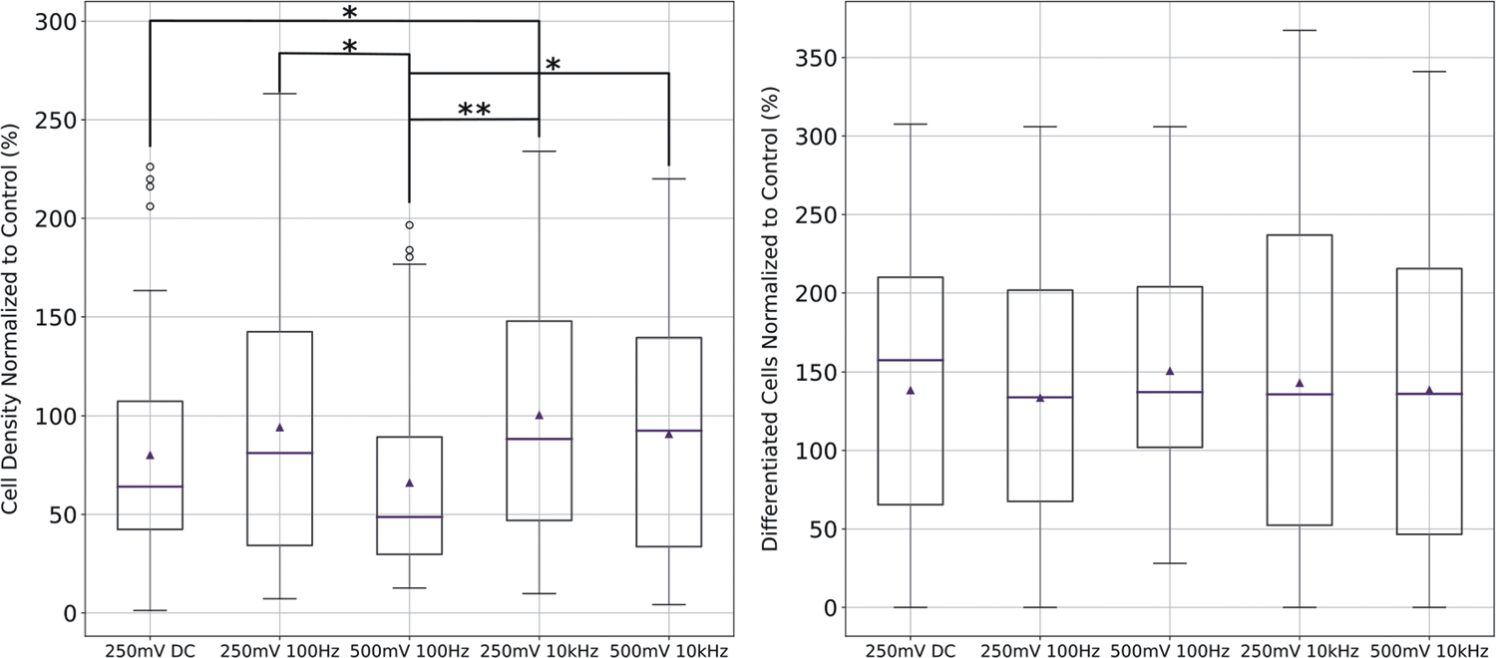
Cell density (left) and differentiation percentage (right) boxplots of the conditions chosen as best. Horizontal lines represent median values, whilst triangles mean values. Outliers are shown as black circles. (*p < 0.05; **p < 0.01; *n* = 60).

Finally, five conditions are going to be analyzed more in-depth in Fig. 5. These conditions have been chosen based on their ability to induce neuronal differentiation. In that regard, although they all seem to perform similarly, the 500mV-100Hz condition is the one with the highest mean value and the least variability. None of these conditions induced a significant increase in proliferation. As can be seen, 250mV-DC and 500mV-100Hz are the conditions with the lowest cell density, being the latter the condition with the lowest mean and median values. This study aims to identify conditions that favor differentiation and reduce cell density, considering the tumorous nature of neuroblastoma cells. The optimal condition was 500mV-100Hz, as it effectively induced differentiation while decreasing cell density. This condition will be further studied via molecular analysis in the next section.

### Cellular and molecular analysis

**Fig. 6:**
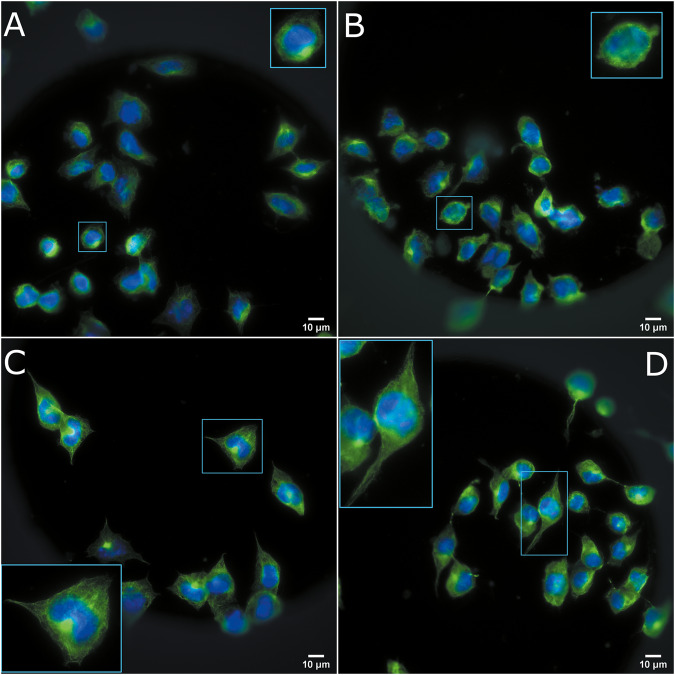
Immunofluorescence images of ES N2a cells. Green represents α-tubulin and blue DAPI. **A** Control with serum, **B** Control without serum, **C** 500 mV–100 Hz with serum, **D** 500 mV–100 Hz without serum. The blue square represents a 2× digital zoom.

To assess the morphology of ES-stimulated N2a cells more thoroughly, we used immunofluorescence to stain one of the most important structural proteins in the cytoskeleton: α-tubulin. This protein is also a fundamental component of neurites. Representative immunofluorescence images of each condition can be seen in Fig. 6. As noted by other authors [14, 24], serum withdrawal has effects on N2a morphology: cells develop cytoplasmic extensions and become less round. The same was true for electrically stimulated cells, both with and without serum; stimulated cells have a more differentiated morphology in general, with more and longer neurites.

**Fig. 7:**
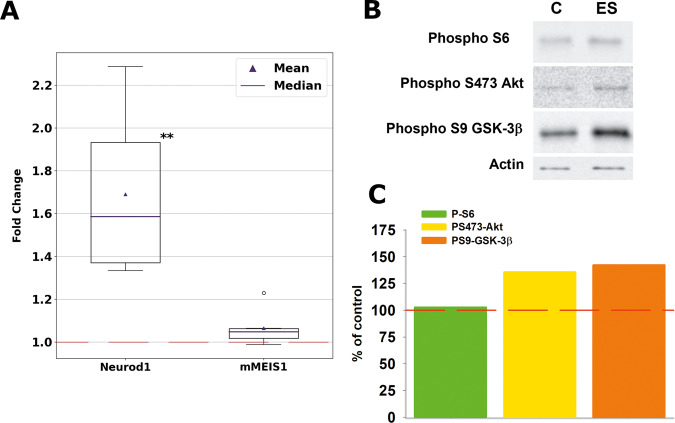
Molecular analysis of N2a cells subjected to 6 hours of ES at 500mV-100Hz. **A** RT-PCR analysis of neuronal differentiation genes. *Neurod1* and mMEIS1 expressions have been normalized to mRplp0 (housekeeping) and then expressed as a fold change referring to the control (red dashed line). (**p < 0.01; *n* = 6). **B** Western blot analysis of proteins involved in cellular pathways regulating neuronal differentiation. **C** Quantification of western blot data. Data are presented as percentage of control (red dashed line). Full and uncropped western blot can be seen in Supplementary Material.

The data shown above seems to indicate that ES induces neuronal differentiation. Therefore, to go deeper into this issue, we performed a molecular analysis in cells subjected to 6 hours of ES at 500mV-100Hz, the optimal condition for neuronal differentiation (see above). First, we analyzed by real-time PCR, the mRNA expression of mMEIS1, a homeobox gene that is associated with cell proliferation in neuroblastoma cells [25, 26], and *Neurod1*, a gene that regulates neuronal differentiation [27, 28]. As shown in Fig. 7A, *Neurod1* was up-regulated in ES cells, however, the expression of mMEIS1 was not affected, suggesting that our ES protocol is mainly promoting neuronal differentiation. Next, we analyzed by western blots the level of phosphorylation of different proteins involved in cellular pathways regulating neuronal differentiation. The phospho-p90RSK and phospho-p44/42 MAPK (Erk1/2) proteins were not detected in N2a cells. However, phospho-ribosomal S6, phospho-S473-Akt, and phospho-S9-GSK-3β proteins were observed (Fig. 7B, C). Interestingly, the ES protocol did not modify the phosphorylation state of the ribosomal S6 protein, but increased phosphorylation of both Akt and GSK-3β, suggesting that ES did not affect the mTOR pathway, but activated the Akt-GSK-3β axis. Taking together, molecular data reveal that ES induces specific molecular responses, that might be regulating the neurites formation previously shown in N2a cells.

## Discussion

This study aims to characterize the sensitivity of N2a cells to ES through their proliferation and differentiation rates. N2a cells serve as both neural precursor and neuroblastoma models. Neural models benefit from higher proliferation in tissue engineering and nerve regeneration, while in neuroblastoma models a high differentiation and low proliferation is desired to suppress tumor growth.

As we have previously reported, ES of 125 mV/mm amplitude increased N2a cell density, for almost every frequency, though the increase in proliferation was not significant in most cases. Other authors have observed enhanced proliferation when stimulating at low amplitudes in bone [29, 30] and neuronal [21, 31] precursor cells, this can be related to the slight increase in proliferation experienced at 125 mV/mm. Furthermore, mammalian skin wounds generate DC fields of 150 mV/mm [32]. Evidence of 1 kHz frequency promoting proliferation has not been found; in [30] 100 Hz was the leading frequency for this parameter, followed by 1 kHz.

According to our results, high amplitude ES caused damage to N2a cells, ultimately leading to cell death, in our opinion, of the disruption of the plasmatic membrane. Amplitudes greater than 750 mV/mm for low frequencies and 1.5 V/mm for high frequencies were the ones that induced cell damage. This behavior was expected due to the electrical nature of N2a cell cultures; the culture and electrodes act as a band-pass filter with its pole at 8 Hz and its zero at 15 kHz, as demonstrated in [33]. This behavior has not been considered in most studies; our electrode configuration, and the fact that we explored a wide range of amplitudes and frequencies supports the electrical properties mentioned.

Our results show that the condition that caused the maximum neuronal differentiation of N2a was a biphasic square wave of 500 mV/mm amplitude and 100 Hz frequency, which also resulted in the minimum cell density for the conditions that did not cause apparent cell damage or death. These results agree with a study by Chang et al. [22]; were a 300 mV/mm–100 Hz monophasic pulse signal enhanced expression of nestin as well as differentiation markers on mouse neural stem and progenitor cells (mNPCs). The authors found that the ES also involved morphological changes resembling differentiation.

Other conditions have been found to induce differentiation of N2a cells, mostly in the range of 250–500 mV/mm. The best frequencies have been found to be DC, 100 Hz and 10 kHz. DC fields of amplitudes in the range of 100–300 mV/mm have been described to induce the most neurite outgrowth in N2a [18]. Other evidence of induction of neuronal differentiation by 10 kHz fields has not been found.

Importantly, we have also shown that the ES protocol of 500 mV–100 Hz is sensed at the molecular level. In addition to morphological alterations, corresponding probably to neurite formation, ES also induced transcriptional up-regulation of *Neurod1* gene. *Neurod1* is a neuronal reprogramming factor, and its expression level is critical for the formation of mature dendritic spines on hippocampal newborn neurons [34], as well as for neuronal reprogramming from both microglia and astrocytes [35]. However, *mMEIS1* expression, a homeobox gene associated with cell proliferation in neuroblastoma cells [25, 26], was not affected, suggesting a specific transcriptional response induced by ES. On the other hand, we have also displayed that the Akt-GSK-3β pathway, but not mTOR, was activated in response to ES. These two cellular pathways are involved in both neuronal proliferation and differentiation. For example, it has been previously shown that the activation of the PI3K/Akt/GSK-3β pathway regulates neuronal proliferation and differentiation in both PC12 and N2a cells [36]. Furthermore, different studies have shown that mTOR plays crucial roles in proliferation, differentiation, and neurite outgrowth [37], however, we did not observe differences in ES compared to control cells. On the other hand, although, preliminary, ES increased the level of phospho-Akt in S473, which is phosphorylated by mTORC2, suggesting that activation of mTORC2 could depend on electrical activity. We are aware that, because of the complexity of the molecular processes underlying neuronal differentiation, we cannot extract any solid conclusion from the present data. Further studies are needed to fully elucidate the molecular pathways activated by ES in relation to neuronal differentiation and proliferation, but our results are premonitory hints of the PI3K/Akt/GSK-3β pathway implications on the neuronal differentiation process through ES.

Due to the electrochemical nature of the gold electrode local environment, it is difficult to separate the effects of the electrical stimulation from those of the redox reactions. Although the average pH in the well did not change, as no color change was induced in the culture medium containing phenol red, we cannot control the microenvironmental conditions in the vicinity of the electrode, where there may be pH changes [38]. The by-products of these redox reactions do not have a toxic effect on the culture, but the effects of the local pH change as a result of them has not been considered. It is also to be noted that at higher frequencies, when the charge delivered is maximum due to the impedance of the electrode, these reactions can be reversed before their by-products leave the electrodes, as the waveform used is charged-balanced [39]. As the authors of [40] affirm, the electrochemical faradic by-products of DC stimulated medium were shown to affect cellular responses; this change might contribute to cell differentiation but also cause cell damage. To reduce electrochemical reactions, they decided to use AC, in this way, electrochemical reactions can be reversed because anode and cathode rotate. However, non-reversible faradic reactions can happen too, so it could be possible that changes in the pH were also affecting cell differentiation.

Many more parameters could have been tested: lower amplitudes, higher frequencies, longer or shorter stimulation times, different waveforms, etc. Other neural precursor cell lines could be used, such as SH-SY5Y or PC12. We plan to extend the study of ES to human neuroblastoma cells as a possible treatment for this type of tumor, due to the effects of the best conditions tested on neuronal differentiation and cell density.

## Conclusions

In this study, N2a cells were subjected to electrical stimulation using biphasic square voltage waves spanning a range of amplitudes (from 125 mV/mm to 1.5 V/mm) and frequencies (from DC to 100 kHz). Special care was taken to ensure that the EF was correctly received by the culture and that the parameters and environment were controlled. N2a cells reacted differently to the various EF applied: low amplitudes seemed to enhance proliferation for most frequencies, especially 1 kHz; high amplitudes produced cell damage that was more pronounced at lower frequencies. Medium amplitudes (250 and 500 mV/mm) enhanced neuronal differentiation. An inverse relation between differentiation and cellular density was found. The 500 mV–100 Hz condition was found to be the optimal to promote neuronal differentiation, as well as to reduce cell density, for that reason it was chosen as the best condition. 500 mV–100 Hz ES is sensed at the molecular level: *Neurod1* expression was up-regulated in cells stimulated with respect to the control. A higher level of phosphorylation was found for Akt and GSK-3β on N2a cells stimulated with the same condition. This suggests that the ES could be activating the Akt-GSK-3β pathway leading to the differentiation process.

## Materials and methods

### Experimental setup

An electrical stimulation system was designed and developed. This system generates the EFs, adapts them for the biological environment, and applies them to the culture; these functions are done by the signal generation circuit, the analog stage (ASE), and the support stage (SUP), respectively. Apart from this, the system has mechanical components that house all of the previously mentioned parts and ensure their correct performance.

#### Electrode configuration

The system is based on 8W10E+ electrode plates (Applied Biophysics, Troy, NY, USA), which are used as electrode-populated culture chambers. These plates have eight wells, each with two sets of 20 interdigitated round gold electrodes with a diameter of 250 µm; the separation between electrodes is of approximately 1 mm, allowing to transform all the voltages directly to V/mm. These plates ensure that the culture receives the generated EF unequivocally, since the application points are uniformly distributed, and the cells rest directly on top of the electrodes surface. This ensures that most field lines are going through the cells on their way to an electrode of the opposite polarity. On the other hand, direct coupling, where electrodes are introduced from the top [19, 21, 41–43], can introduce uncertainty regarding the field experienced by the cells, as the field lines traverse not only the culture vicinity and the cells itself, if the frequency of EF allows it, but also the region above it. A COMSOL simulation of the current distribution in both setups was performed to confirm all of the above (Supplementary Fig. 1).

#### Electronic circuits

The signal generation is carried out by a Digital-To-Analog Converter (DAC) inside a microcontroller (MCU); we have used the STM32L4R5ZI (STMicroelectronics, Geneva, Switzerland) in its development board (Nucleo-L4R5ZI). The electrical stimulation signal is updated by the DAC using direct memory access (DMA); the MCU shuts down after the programmed stimulation time is over. The MCU is powered at 3.3 V, since the waves wanted are bipolar, the signals generated are centered at 1.65 V and then, this voltage is subtracted by a differential amplifier in the ASE. In order to have access to that voltage and make it the same for the MCU and the ASE, a reference IC (REF1933, Texas Instruments, Dallas, TX, USA) was used.

**Fig. 8:**
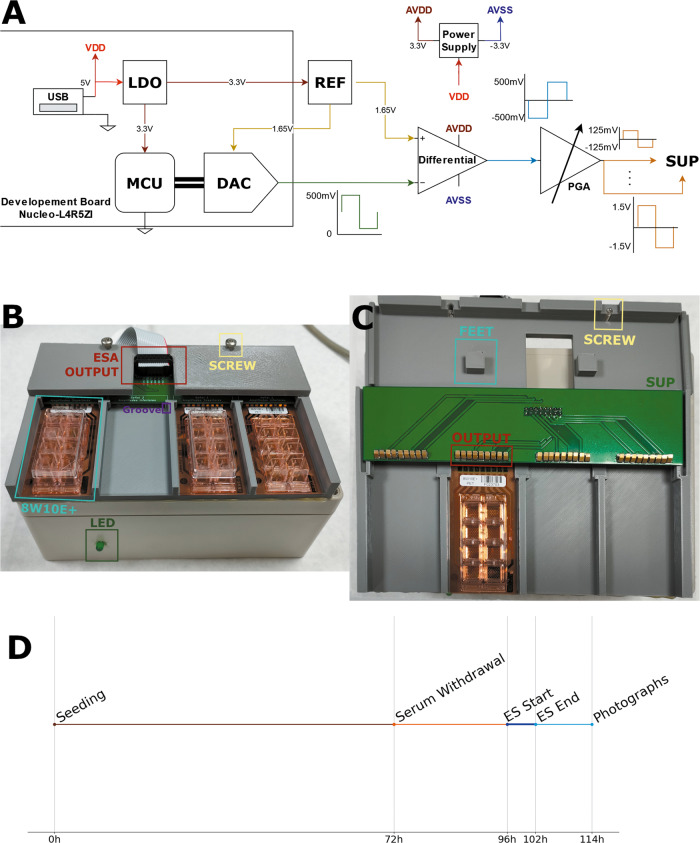
Electrical stimulation system and stimulation protocol overview. **A** Diagram of the generation of the voltage stimulation signals. **B** Outside view. **C** Detailed view of the SUP and mechanical components, the PCB has been flipped for display. **D** Stimulation protocol timeline.

In the ASE, the signal generated by the DAC is inverted and re-centered at 0 V by an operational amplifier (OPA4172, Texas Instruments) in differential configuration. An operational amplifier, in inverting configuration, returns the signal to its correct polarity and modifies its amplitude; this block functions as a programmable gain amplifier (PGA). The gain of this amplifier can be configured by the resistor ratio of the inverting configuration. The signal is correctly conditioned for the load of the culture plus the electrodes and has passed simulations of extreme load conditions (R_L_ = 100Ω and C_L_ = 2nF). The ASE has its own dual rail power supply to reduce possible artifacts and noise in the final signals. The signal generation process and the output of every stage can be seen in Fig. 8A.

#### SUP

Lastly, the SUP ensures that the signals are received correctly by the 8W10E+ plates and that every signal goes to the right well. The SUP PCB ensures the connection between the ESA and the contacts of the electrode plate. Two mechanical components have been designed and 3D-printed for that purpose. The bottom part holds the PCB and four 8W10E+ plates, the top part ensures that the contacts of the PCB are tightly pressed against the ones on the electrode plate. It does so by using two screws and feet that push the PCB downwards. The plates are inserted into the channels of the bottom part and then pushed to meet the connector in the PCB, the pressure form the connectors and a groove in the channel keeps them from moving (Fig. 8B, C).

The ES system designed and developed allows for four plates to be stimulated at the same time, with a maximum of two different signals (waveform and frequency) each with 8 different amplitudes.

### Experimental and electrical stimulation protocol

N2a cells were stimulated 24 h after serum withdrawal, which was 72 h after seeding. 18 h after the ES ended, a timeline of this procedure can be seen in Fig. 8D. The effects of amplitude and frequency are going to be studied, fixing the waveform and the stimulation time. The applied amplitudes range from 125 mV/mm to 1.5 V/mm and the frequencies from DC (0 Hz)–100 kHz. ES duration is fixed to 6 h according to previous studies [18, 19, 44], where cells were stimulated for 2–8 h. The waveform chosen is a biphasic square wave with a duty cycle of 50%, as seen in Supplementary Fig. 2. Pulsatile waves are the most commonly used AC signals [1, 9, 42, 45–52]. Monopolar waves accumulate electrical charge, due to the constant direction of the current; in our case, the ES waveform used is bipolar, which balances the electrical charge.

### Cell culture

N2a cells were cultured following the method previously described in [53] being the only difference the number of cells seeded. In this study, 2000 cells were seeded in 500 µL of completed medium in each of the wells of the 8W10E+ plate. This lower initial density allowed for a better environment for N2a differentiation and avoided confluency problems under conditions with greater proliferation. N2a cells remained seeded for 3 days before changing to serum-free medium, after which ES was applied. Cells were kept at 37 °C in a humidified atmosphere with 5% CO2 throughout the duration of the experiments.

### Cell counting method

18 h after ES had ended, images were taken with a 20× objective in an inverted microscope (Leica, Wetzlar, Germany). In order to obtain cell differentiation percentage and cell density, differentiated and non-differentiated cells on top of the electrode were counted using the method and tools described in [54]. Cell density was calculated as the total number of cells counted on top of the electrode divided by the surface area of the electrode in mm^2^. Differentiation percentage was calculated as the number of differentiated cells divided by the number of total counted cells. More than 6000 images were counted for this study.

### Immunofluorescence assay

2000 N2a cells were seeded in each well of an 8W10E+ plate as previously described. The next day, 4 of the wells had their medium refreshed with completed medium and the other 4 with serum-free medium. 24 h later, 2 wells with and 2 without serum were stimulated for 6 h at 500 mV/mm–100 Hz. The following day, the immunofluorescence protocol presented in [53] was carried out, with the exception that the primary antibody was diluted 1:5000 to ensure optimal visibility through the electrode. Images were taken in a fluorescent inverted microscope with a 63× objective (Leica).

### RT-PCR assay

The expression analysis was carried out under the 500 mV–100 Hz condition. RNA was isolated from four control, and four ES wells, performed in duplicate, 24 h after ES started, using the E.Z.N.A. Total RNA Kit I (Omega Bio-Tek. GA, USA). Total RNA was reverse transcribed with the iScript cDNA Synthesis Kit (Bio-Rad. CA, USA). The real-time PCR amplification was done in a CFX Connect Real-Time System (Bio-Rad) using SYBRGreen (Bio-Rad), and the cDNA obtained as template. The genes amplified were *Neurod1*, mMEIS1, and mRplp0 (sequences in Supplementary Material). *Neurod1* and mMEIS1 expression was normalized with respect to mRplp0 expression, a ribosomal gene commonly used as housekeeping. Each specific gene was determined in triplicate for each experiment. Primers were generously provided by Dr. Mario García Domínguez [55].

### Western blot analysis

Western blots were performed as previously described [56]. Nine micrograms of proteins of control and ES cells (500 mV–100 Hz) were loaded on a 12% polyacrylamide gel for electrophoresis (SDS–PAGE; Bio-Rad) and transferred to a nitrocellulose membrane (Hybond-C Extra; Amersham, Barcelona, Spain). Membranes were blocked for 1 h, and finally incubated overnight at 4 °C, with the following primary antibodies: (i) rabbit polyclonal PathScan® Multiplex Western Cocktail antibodies against Phospho-p90RSK, Phospho-Akt, Phospho-p44/42 MAPK (Erk1/2) and Phospho-S6 Ribosomal (#5301 Cell Signaling, Danvers, MA, USA); (ii) rabbit polyclonal antibody against Phospho-GSK-3β (Ser9) (#9336 Cell Signaling, Danvers); and (iii) mouse monoclonal antibody against β-actin (A1978 Merck Life Science S.L.U. Madrid, Spain). After that, membranes were incubated with the appropriate anti-rabbit or anti-mouse secondary antibody (Dako) horseradish peroxidase-conjugated, at a dilution of 1/5000 and developed using the ECL-plus detection method (Amersham) and the ImageQuant LAS 4000 MINI GOLD (GE Healthcare Life Sciences, Barcelona, Spain). For quantification, the optical density of individual bands was analyzed using the ImageJ software (NIH, USA), and the optical density of each band was normalized relative to the optical density of β-actin.

### Statistical analysis

All statistical analyses were performed using one-way analysis of variance (ANOVA) test, where *p* values < 0.05 were considered statistically significant. Unless otherwise noted, all tests have been performed with reference to the control.

### Supplementary information

Original Western Blot

Supplementary Material

Supplementary Figure 1

Supplementary Figure 2

## Data Availability

The datasets used during this study are available on reasonable request.
