# Supplementary figures and images for: Electrical pulse stimulation parameters modulate N2a neuronal differentiation
Source: Cell Death Discov. 2024 Jan 25;10:49. doi: 10.1038/s41420-024-01820-y (PMC10810886; doi:10.1038/s41420-024-01820-y)

ACTIN

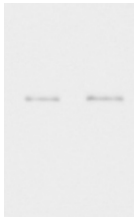

PS473Akt

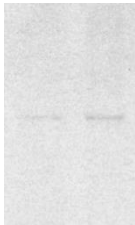

PhosphoGSK3B

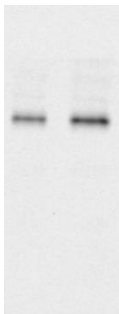

PS6

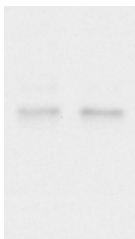

Supplement: Supplementary file 1 — Original Western Blot [file 41420_2024_1820_MOESM1_ESM.pdf]

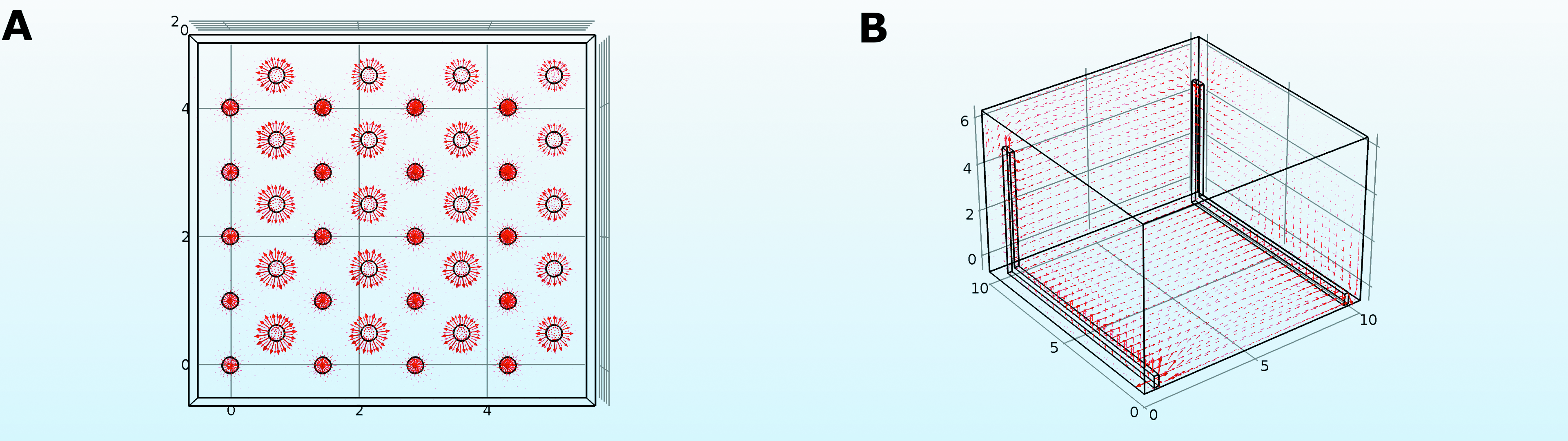

Supplement: Supplementary file 3 — Supplementary Figure 1 [file 41420_2024_1820_MOESM3_ESM.tif]

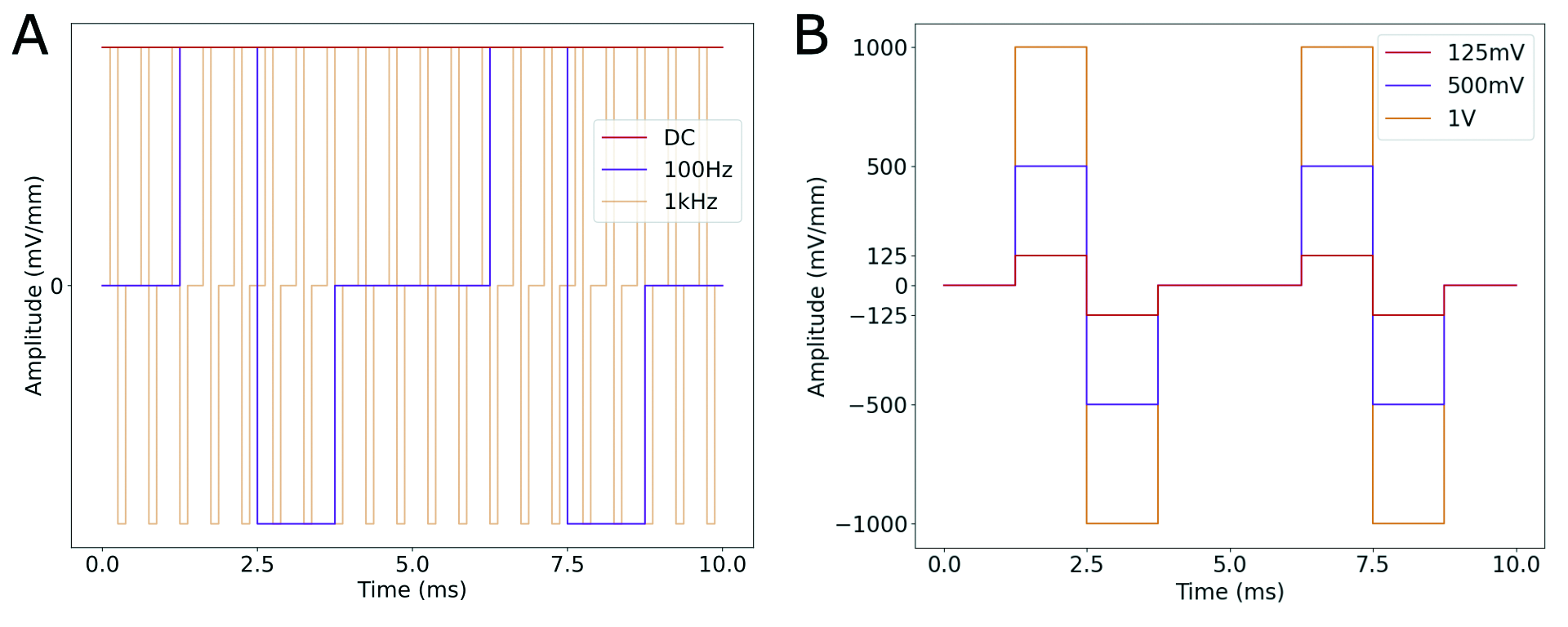

Supplement: Supplementary file 4 — Supplementary Figure 2 [file 41420_2024_1820_MOESM4_ESM.tif]
