# Supplementary material for: Electrical pulse stimulation parameters modulate N2a neuronal differentiation
Source: Cell Death Discov. 2024 Jan 25;10:49. doi: 10.1038/s41420-024-01820-y (PMC10810886; doi:10.1038/s41420-024-01820-y)
Supplement: Supplementary file 2 — Supplementary Material [file 41420_2024_1820_MOESM2_ESM.docx]

**Supplementary Figures**


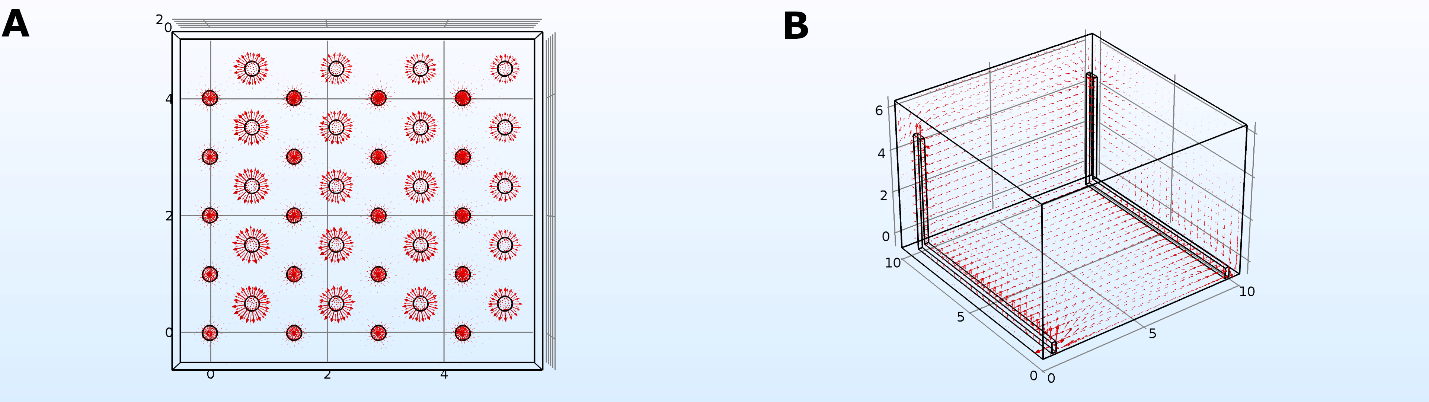


Supplementary Figure 1: Current density COMSOL Simulations. A: 8W10E+. B: Direct coupling setup example. Current is shown as red arrows proportional to the value. Arrows have been scaled for better visualization. Materials used are gold for the electrodes and DMEM (60ε, 0.5 S/m) for the environment.


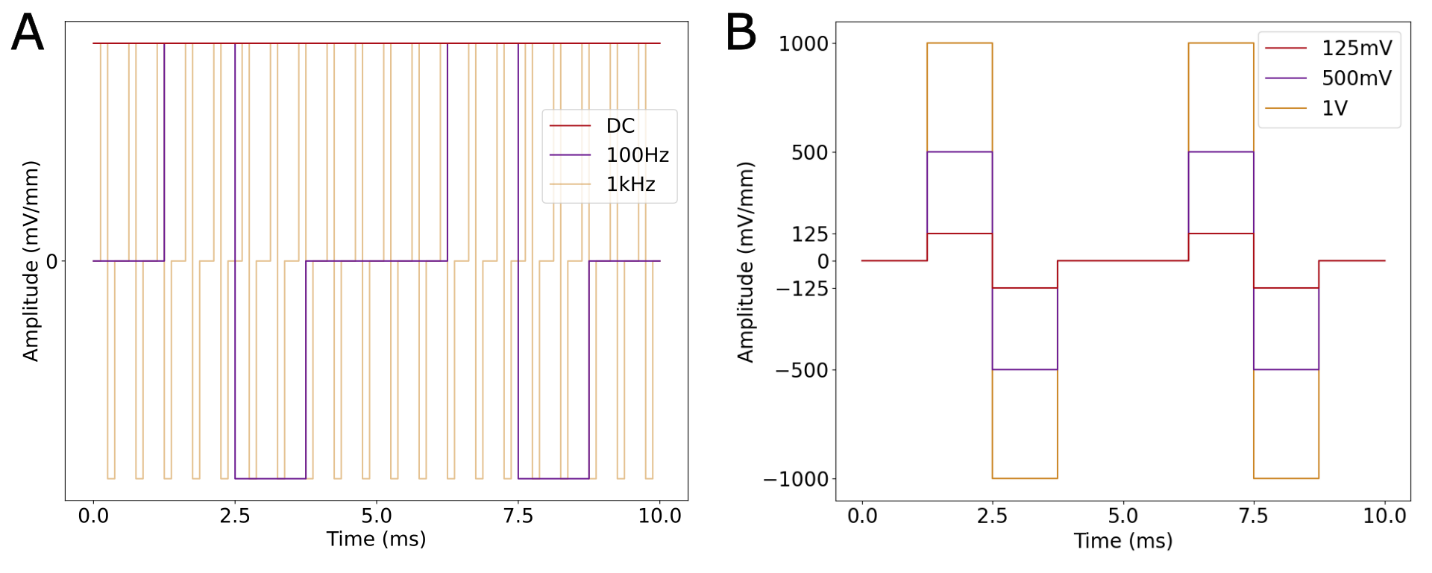


Supplementary Figure 2: A: Example of the electrical stimulation signals outputted by the ES system and applied to the culture. A: Three different frequencies: DC, 100Hz and 1kHz. B: Three different amplitudes: 125mV/mm, 500mV/mm and 1V/mm at 100Hz.

Primer Sequences

Primers used had the following specific primer pairs: from 5´to 3´:

***Neurod1*** forward: TCAGCATCAATGGCAACTTC and reverse: AAGATTGATCCGTGGCTTTG.

**mMEIS1** forward: CTTTCCCAAAGTAGCCACCA and reverse TGTGCCAACTGCTTTTTCTG.

**mRplp0** forward: CCAGGCTTTGGGCATCAC and reverse CTCGCTGGCTCCCACCTT.
